# Effect of Aging Temperature on the Microstructure and Properties of Economical Duplex Stainless Steel

**DOI:** 10.3390/ma12132085

**Published:** 2019-06-28

**Authors:** Hongliang Xiang, Chunyu Liu, Liping Deng, Kaikui Zheng

**Affiliations:** 1 School of Mechanical Engineering and Automation, Fuzhou University, Fuzhou 350116, China; 2 Jinjiang Science and Education Park, Fuzhou University, Jinjiang 362251, China

**Keywords:** aging treatment, tensile strength, corrosion resistance, antibacterial properties, precipitated phases

## Abstract

In this study, NSSC 2120 economical duplex stainless steel was prepared and the effects of aging temperatures on its intermetallic phase morphology, tensile strength, elongation, corrosion resistance, and antimicrobial properties were investigated. The results revealed that after aging the sample at 650 °C, it exhibits better pitting corrosion resistance and higher tensile strength. Upon increasing the aging temperature up to 750 °C, the pitting corrosion resistance and tensile strength of the samples were decreased due to the precipitation of the ε-Cu phase in the matrix. Moreover, with the further increase in the aging temperature to 850 °C, oxides containing Mn and Cr (CrMn_1.5_O_4_) and sulfides (MnS) precipitated from the samples, further decreasing their pitting corrosion resistance and tensile strength. Upon aging the samples at 950 °C, no second phase was observed and the corrosion resistance was less than that of the sample after aging at 650 °C, but the tensile strength was greater than that of the sample after aging at 650 °C. Antibacterial test results revealed that the sample after aging at 750 °C exhibited a good antibacterial effect due to the precipitation of the rod-shaped ε-Cu phase.

## 1. Introduction

Duplex stainless steel exhibits the excellent toughness and weldability of austenitic (γ) stainless steel and the high strength, chloride resistance, and stress corrosion of ferritic (α) stainless steel; hence, duplex stainless steel is widely employed in the petroleum and energy industries, marine engineering, and other fields [1,2,3,4,5]. In recent years, due to the high cost of Ni, which is a key element in duplex steel, the manufacturing cost of duplex stainless steel has increased considerably. Some studies have reported that the substitution of Mn and N for Ni can significantly decrease production costs, which has become the main research direction [6,7]. However, isothermal aging at temperatures in the range 600–1000 °C or inadequate heat treatment leads to the precipitation of secondary phases such as nitrides, σ phases, χ phases, and other intermetallic phases [8,9,10], which will in turn significantly reduce material performance [11]. For example, Straffelini et al. [12] investigated the effect of aging treatment on the impact energy of economical duplex stainless steel. Results revealed that soaking samples at 550–850 °C for 5–120 min leads to the observation of precipitated phases at the α/α and α/γ phase boundaries, which decreases the impact energy and brittle fracturing. Zhang et al. [13] investigated the effect of aging on the corrosion resistance of 2101 economical duplex stainless steel and reported that the pitting potential and critical pitting temperature of the samples sharply decreased after soaking the samples at 700 °C for 30 min, and the corrosion resistance of the materials dramatically decreased. Guo et al. [14] investigated 2304 lean duplex stainless steel (LDSS) and revealed that after the sensitization of 2304 LDSS at 700–750 °C for 2 h, Cr_2_N predominantly precipitated in the intergranular phase, thereby decreasing materials’ local corrosion resistance. Currently, most studies on the changes in the structure and properties of economical duplex stainless steel after aging have mainly focused on UNS S2101 and UNS S2304 duplex stainless steel. In recent years, compared to conventional 304 austenitic stainless steel, new NSSC 2120 economical duplex stainless steel has been reported to exhibit better mechanical properties and corrosion resistance [15,16]. However, relevant studies regarding the effect of aging on its structure, mechanical properties, resistance, and antimicrobial properties have not yet been reported. With this background, NSSC 2120 economical duplex stainless steel has been prepared and the effects of the aging temperature on its structure and related properties has been investigated to guide the theoretical research into and practical applications of materials.

## 2. Materials and Methods

NSSC 2120 cast stainless steel was prepared in a medium-frequency induction furnace (Model SPZ-35, Shuangping Power Supply Technologies Co., Ltd., Shenzhen, China) with 316L stainless steel, metal Cr, ferromolybdenum, electrolytic nickel, and chromium nitride alloy as the raw materials. Table 1 summarizes the chemical composition of NSSC 2120 cast stainless steel as measured using a direct reading spectrometer (Model ARL3460, ARL Corp., Bern, Switzerland).

Heat treatment involved a solid-solution process and aging treatment. The solid-solution process was carried out at 1150 °C for 2 h, followed by quenching with water, and aging was carried out at 650, 750, 850, and 950 °C for 2 h, followed by cooling in air. Hereafter, the samples will be referred to as SX-650, SX-750, SX-850, and SX-950, respectively. The final heat-treated samples were polished to a mirror-like finish using 120–3000# sandpaper and etched using Murakami’s solution. Finally, metallographic images were recorded using an optical microscope (Model BM2100 POL, Jiangnan Novel Optics Co., Ltd., Nanjing, China). Then, 0.3 mm thin-film samples were cut from the SX-650, SX-750, SX-850, and SX-950 samples and ground into 0.05 mm thick smooth surface slices. The specimens were then electrolytically sprayed (with a mixture of 10% perchloric acid and 90% ethanol) and thinned (at 12 V and 0 °C). Transmission electron microscopy (TEM) images were recorded with a microscope (Model TECNAI G2F20, FEI Company, Hillsboro, OR, USA) to observe the morphology and detect the chemical composition.

The final heat-treated samples were processed into tensile samples according to the ISO 6892-1-2009 standard. Tensile tests were carried out on a universal material testing machine (Model CMT-5105, New Sice Materials Testing Co., Ltd., Shenzhen, China) at a tensile rate of 3 mm/s. The samples’ pitting corrosion resistance was estimated using an electrochemical workstation (Model CHI660E, CH Instruments Inc., Shanghai, China) with natural seawater as the corrosion medium. The temperature of the experimental system was controlled at (25 ± 1 °C) using a constant-temperature water bath (Model HH-2, Jiangnan Instrument Factory, Jintan, China). The antimicrobial properties of the samples were examined using a constant-temperature incubator (Model GNP-9080, Jinghong Experimental Equipment Co., Ltd., Shanghai, China) and a constant-temperature oscillation box (Model ZWY-2102C, Zhicheng Analytical Instrument Manufacturing Co., Ltd., Shanghai, China). Each measurement was carried out in triplicate and the results were then averaged.

## 3. Results and Discussion

### 3.1. Effect of Aging Temperature on the Microstructure

**Figure 1:**
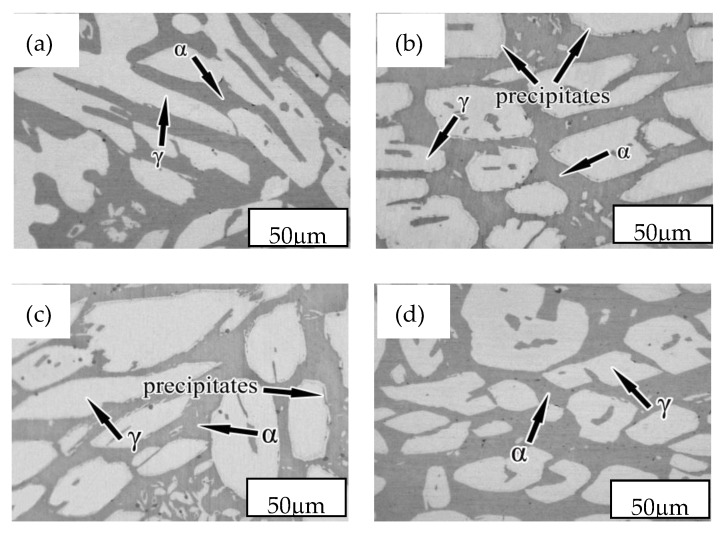
Microstructures of NSSC 2120 after aging at different temperatures: (**a**) SX-650; (**b**) SX-750; (**c**) SX-850; (**d**) SX-950.

Figure 1 shows the microstructure of the NSSC 2120 specimens after aging at different temperatures.

**Figure 2:**
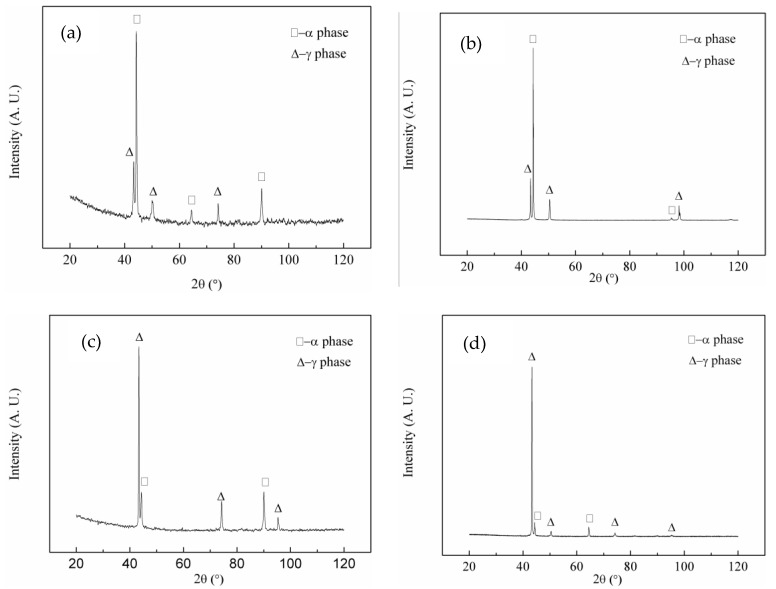
XRD patterns of NSSC 2120 after aging at different temperatures: (**a**) SX-650; (**b**) SX-750; (**c**) SX-850; (**d**) SX-950.

The microstructure was comprised of a dark gray ferrite phase (α) and a light white austenite phase (γ) (Figure 1). Precipitates were not observed for the SX-650 or SX-950 samples. Black precipitates were observed at the interface between the two phases and in the α-matrix (Figure 1b,c). To determine the precipitate structure, X-ray diffraction was employed for sample analysis (Figure 2).

**Figure 3:**
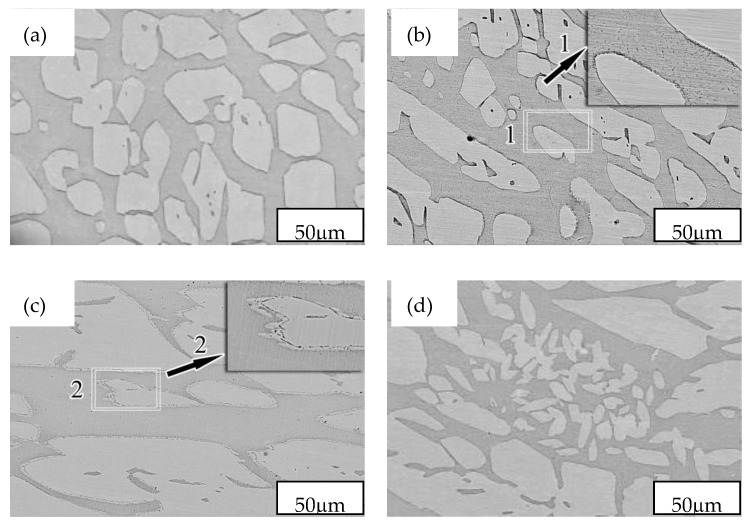
SEM micrographs of NSSC 2120 after aging at different temperatures: (**a**) SX-650; (**b**) SX-750; (**c**) SX-850; (**d**) SX-950.

Only two diffraction peaks, one each corresponding to the α and γ phases, were observed for the SX-650, SX-750, SX-850, and SX-950 samples, with no diffraction peaks corresponding to the second phase (Figure 2). This observation was probably related to the small number of precipitates and the absence of diffraction peaks corresponding to the precipitates. To further investigate the precipitates, the aged samples were investigated by scanning electron microscopy (SEM, Figure 3).

**Figure 4:**
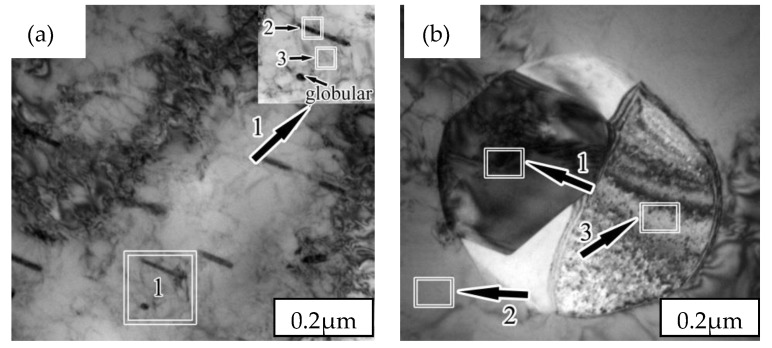
TEM images of samples after aging at 750 and 850 °C: (**a**) SX-750; (**b**) SX-850.

A second phase was not observed in the SEM images of the SX-650 and SX-950 samples (Figure 3). This result was in agreement with those reported by metallographic observation and XRD diffraction peaks. Black precipitates were observed in the α-matrix of the SX-750 sample (Figure 3b), and the zone 1 enlargement map clearly revealed dotted and linear precipitates. The zone 2 enlargement map in Figure 3c clearly revealed the presence of precipitates of the SX-850 samples at the boundary between the α and γ phases. TEM images of the SX-750 and SX-850 samples were recorded to further analyze the precipitates (Figure 4).

**Figure 5:**
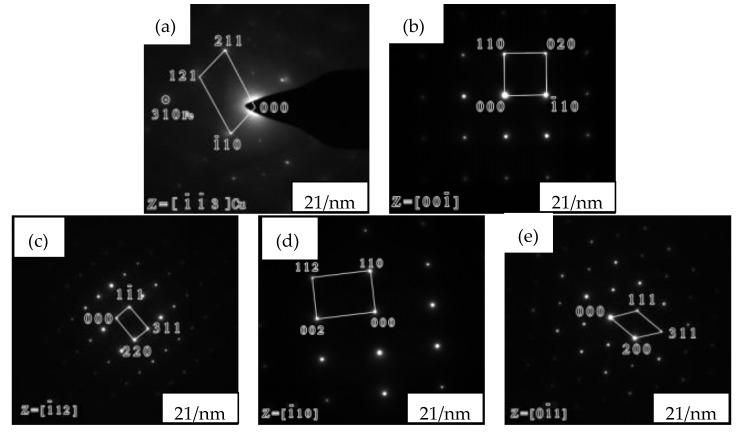
The selected diffraction spots of SX-750 and SX-850: (**a**) SX-750, region 2; (**b**) SX-750, region 3; (**c**) SX-850, region 1; (**d**) SX-850, region 2; (**e**) SX-850 region 3.

By the magnification of zone 1 in Figure 4a, a majority of rod-shaped black precipitates were observed for the SX-750 specimens (rod length of ~340 nm and width of ~30 nm), in addition to the distribution of a few spherical precipitates (diameter of ~40 nm), possibly corresponding to the cross-section of the short rod. Irregular precipitates were observed for the SX-850 specimens. The energy dispersive spectrometer (EDS) analysis of zones 2 and 3 of the SX-750 sample and zones 1, 2, and 3 of the SX-850 sample (numbered 1, 2, 3, 4, and 5, respectively) was carried out (Table 2). Zone 2 of the SX-750 sample was rich in copper; zone 1 of the SX-850 sample was rich in O, Cr, and Mn; and zone 3 of the SX-850 sample was rich in Mn and S. EDS analysis revealed that zone 2 of the SX-750 sample was possibly a copper-rich phase, while zones 1 and 3 of the SX-850 sample were possibly oxides and sulfides, respectively. To further determine the type of precipitates, selective diffraction analysis was carried out for zones 2 and 3 of the SX-750 sample and zones 1, 2, and 3 of the SX-850 sample. Figure 5 shows the diffraction spots of the SX-750 and SX-850 samples.

The analysis of the diffraction spots (Figure 5a) revealed a short rod phase exhibiting a face-centered cubic (fcc) structure that is indicative of the ε-copper phase according to the chemical composition summarized in Table 2. By combining the analysis of the other diffraction spots and EDS results, an α phase with a body-centered cubic (bcc) structure, a CrMn_1.5_O_4_ phase with an fcc structure, an α phase with a bcc structure, and sulfides (MnS) with an fcc structure were observed in Figure 5b,c,d, respectively. The precipitation of the ε-copper phase in the α-matrix after aging at 750 °C was related to the following reasons: First, the solubility of copper in the α matrix was low. Second, the low density of the α-matrix meant that copper was easily supersaturated and it rapidly diffused in the α matrix, which was conducive to its nucleation and growth in the α matrix [17]. In addition, the presence of dislocations in the γ matrix was conducive to the migration of copper atoms to α. In addition, ε-Cu in SX-750 was a steady-state Cu-rich phase rather than a metastable Cu-rich phase, as determined by diffraction spot analysis; this result was confirmed by the size profile in the TEM image (Figure 4a and Figure 5a). Previously [18,19], a metastable copper-rich phase was reported as growing to 1.6 nm and then converting into a stable copper-rich phase with an fcc structure. Meanwhile, the size of the copper-rich phase in our experiment was considerably greater than 1.6 nm.

The precipitation of MnS was mainly controlled by the diffusion of the Mn atom because the diffusion coefficient of Mn was considerably less than that of S at the same temperature [20]. Through low-temperature aging, the diffusion of supersaturated Mn atoms was slow and it was difficult to precipitate MnS. However, with the increase in temperature, Mn diffused a short distance to promote the precipitation of MnS at crystal dislocations and grain boundaries [21,22], so MnS was precipitated from the SX-850 sample. In addition, some studies have reported that the aging of Fe-Cu-Ni-Mn quaternary alloys led to the precipitation of the Mn-rich region around the Cu-rich phase [23]. At an extremely high aging temperature, the enrichment area of Mn did not disappear as the temperature increased. Meanwhile, O and S dispersed in the steel diffused to the Mn-rich area, which afforded Mn-containing oxides (CrMn_1.5_O_4_) and sulfides (MnS). Oikawa et al. [24] suggested that MnS tends to nucleate on oxides, which is consistent with the experimental results.

### 3.2. Effect of Aging Temperature on the Mechanical Properties

**Figure 6:**
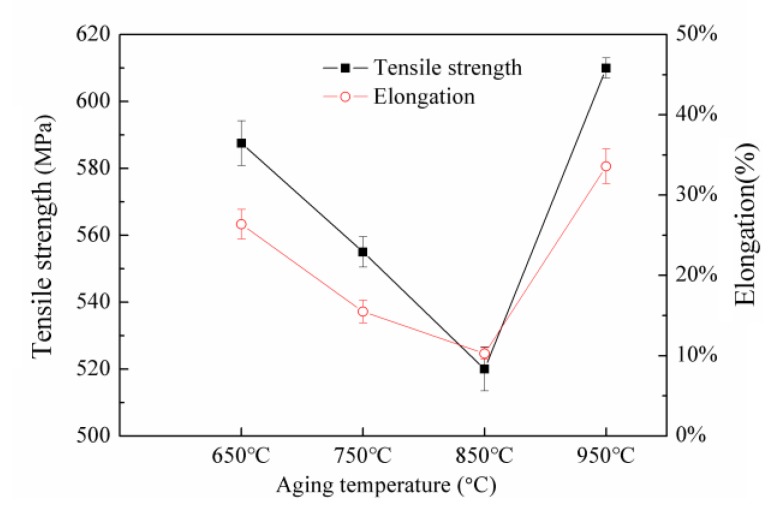
Tensile strength and elongation of NSSC 2120 after aging at different temperatures.

Figure 6 shows the tensile strength and elongation of NSSC 2120 duplex stainless steel after aging at different temperatures. The tensile strength and elongation of the materials clearly decreased as the temperature increased from 650 to 850 °C, exhibiting the lowest strength and elongation when the temperature was increased to 850 °C (Figure 6).

**Figure 7:**
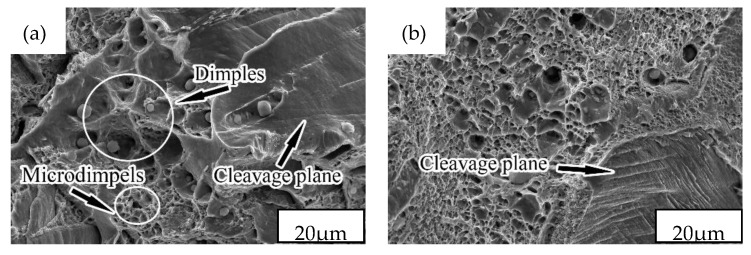

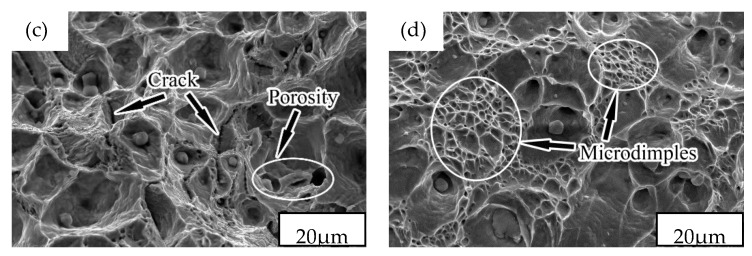
Fracture morphologies of NSSC 2120 after aging at different temperatures: (**a**) SX-650; (**b**) SX-750; (**c**) SX-850; (**d**) SX-950.

With the increase in the temperature to 950 °C, the tensile strength and elongation increased considerably and reached their peak value. To further analyze the above results, each specimen’s tensile fracture morphology was observed and analyzed (Figure 7).

The fracture morphology of SX-650 revealed dimples and cleavage planes (Figure 7a). A large dimple exhibited a certain depth of field, but the fracture exhibited a large cleavage plane, revealing that the material’s tensile strength was “compromised” and does not exhibit the best performance. Large, flat cleavage surfaces (as indicated by arrow 7b) were observed in the cross-section of the specimen at 750 °C. Few large and deep dimples were observed, and an increased number of dimples were observed as aggregated micropores. Under external forces, the aggregated micropores were torn apart before their aggregation and growth. The whole fracture was shaped like a “flat block.” From fracture analysis, the specimen’s tensile strength could be considered less than that of SX-650. In addition, previous studies [25] have reported that dislocation hindrance is weakened because the size of the precipitated phase is > 40 nm. However, the size of ε-Cu in this experiment was considerably greater than this value, which indicates that the tensile strength of the SX-750 specimen was not ideal. The fracture morphology of the SX-850 specimens revealed few cleavage planes, several large and deep dimples, connected holes, and several clear cracks (as indicated by arrow 7c). This result indicates that the material’s tensile strength of is poor and the material would fracture under low stress. Hence, the precipitated CrMn_1.5_O_4_ and MnS non-metallic inclusions in the sample possibly split the matrix and decreased the effective cross-sectional area of the steel. In addition, under the action of external forces, the precipitated phase hindered the dislocation movement, which led to the stress concentration caused by the dislocation pile-up group. Increasing the stress to a certain extent initiated crack source and cracking would occur that significantly affected the materials’ tensile strength. Compared to the sample after aging at 650 °C, several aggregation zones with a dimple pattern were observed on the fractured surface of SX-950 (Figure 7d). In addition, its dissociation surface was small and its tearing edge was short, which indicate that its performance was better than that of the former. In addition, studies [26,27] have revealed that when dimples aggregate and grow under external forces, their interconnection ability is relatively enhanced and the tensile strength improves, which is consistent with the experimental results.

### 3.3. Effect of Aging Temperature on the Pitting Corrosion Resistance

**Figure 8:**
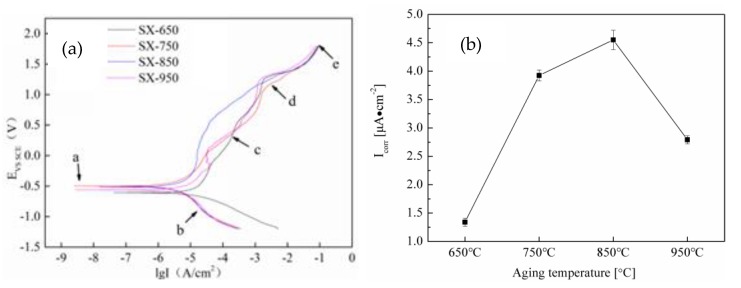
The polarization curves and corrosion current density of NSSC 2120 after aging at different temperatures: (**a**) Polarization curves; (**b**) corrosion current density.

The electrochemical nature of metal corrosion involves the formation of corrosive galvanic cells. Figure 8 shows the polarization curves and corrosion current density of NSSC 2120 after aging in seawater at different temperatures. In Figure 8a, there seems to be little difference in the self-corrosion potential among the samples after aging at different temperatures, which indicates that the samples have similar corrosion tendency.

**Figure 9:**
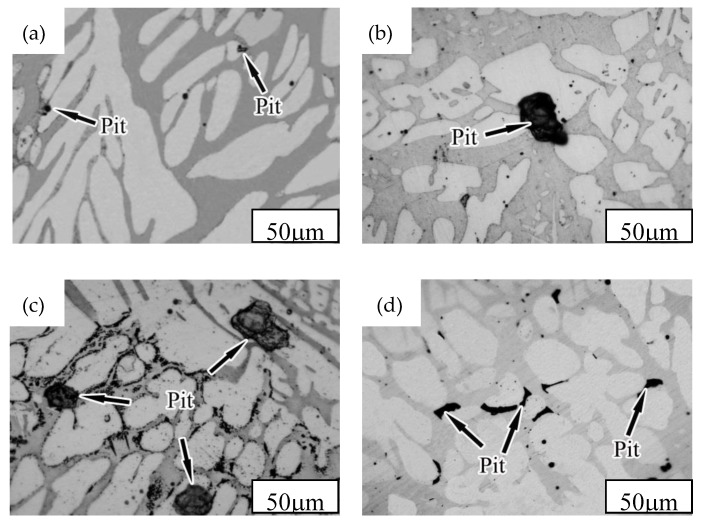
The pitting morphologies of NSSC 2120 after aging at different temperatures: (**a**) SX-650; (**b**) SX-750; (**c**) SX-850; (**d**) SX-950.

According to Faraday’s law, the corrosion rate of metals is proportional to the corrosion current density (Icorr) [28]; hence, Icorr can be used to characterize materials’ pitting corrosion resistance. As the temperature increased, Icorr first increased and then decreased (Figure 8b). For the SX-650 and SX-850 samples, the Icorr values were 1.34 × 10^−6^ μA·cm^−2^ (lowest) and 4.55 × 10^−6^ μA·cm^−2^ (highest), respectively. To further investigate the corrosion mechanism of aged NSSC 2120, the pitting morphology of NSSC 2120 was investigated by SEM (Figure 9).

A small number of pits and pit size were observed for the SX-650 samples, and as the temperature increased to 750 °C, the size of pits increased. When the temperature increased to 850 °C, the highest number of pits and pit size were observed, followed by the decrease in the number and size of pits as the temperature increased to 950 °C (Figure 9). Overall, the change in the regulation of the pitting pit agreed with the Icorr change trend. As the temperature increased from 650 to 750 °C, the pitting corrosion resistance of the specimens dramatically decreased, which corresponded to the precipitation of the ε-Cu phase. First, from the analysis shown in Figure 4, an ~340 nm long rod-shaped ε-Cu phase was observed for the SX-750 sample. Previous studies [29] have revealed that the rod-shaped ε-Cu phase made it difficult to passivate the aged sample after pitting corrosion, thereby decreasing the passivation film’s protective effect on the sample. Second, the difference between the corrosion potential of the ε-Cu phase and the passivation film meant that galvanic corrosion and corrosion were observed. The corrosion rate of the ε-Cu phase with low corrosion potential was increased, while the higher corrosion potential of the passive film decreased. The ε-Cu phase in the passive film became discontinuous due to the dissolution of the galvanic corrosion, leading to a decrease in the sample pitting resistance [30]. Third, the ε-Cu phase easily became the initiation point for pitting corrosion, thereby weakening the sample’s pitting resistance [31]. With the further increase in the temperature to 850 °C, poor-Cr regions that corresponded to the production of CrMn_1.5_O_4_ were observed. The relatively weak passive film around CrMn_1.5_O_4_ was not rapidly repaired after pitting corrosion, which led to an increase in the corrosion current density. In addition, because MnS can rapidly dissolve in a Cl^−^-rich environment and form corrosion products that acidify its local environment and cause a relatively blocked environment, the self-catalytic effect leads to the dissolution of the surrounding matrix and subsequent pitting corrosion [32]. With the increase in the temperature to 950 °C, the pitting corrosion resistance of specimens was better than those of SX-750 and SX-850 as other phase precipitates were not observed, which led to the decrease in the corrosion resistance. However, the pitting corrosion resistance of SX-950 was weaker than that of SX-650, mainly due to the higher content of corrosion-resistant austenite in SX-650, and the relative balance of the two phases was also conducive to the improvement of corrosion resistance.

### 3.4. Effect of Aging Temperature on Antibacterial Properties

**Figure 10:**
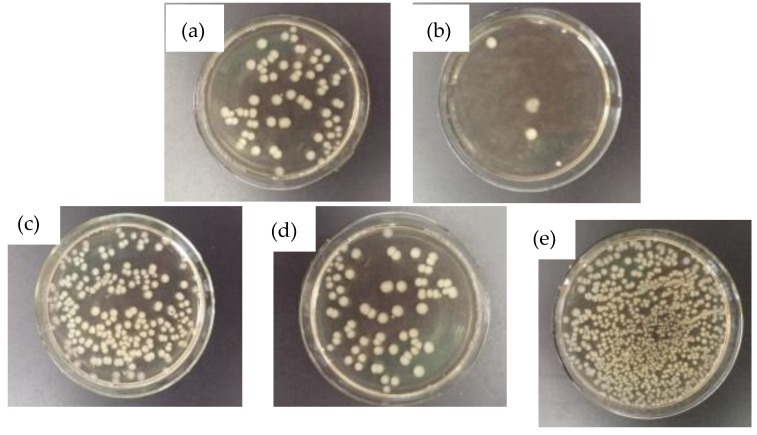
The bactericidal effect of maps of the samples after 24 h interaction with the bacterial solution: (**a**) SX-650; (**b**) SX-750; (**c**) SX-850; (**d**) SX-950; (**e**) 2205.

According to the antimicrobial Japanese industrial standards JIS Z:2801:2000 and Chinese industrial standard QB/T2591-2003, the antimicrobial properties of antimicrobial-treated materials at different temperatures were compared and analyzed by the coating method. The selected reference material was 2205 duplex stainless steel with no antimicrobial effect, and Gram-negative *Escherichia coli* was selected as the test strain. Figure 10 shows the bactericidal efficacy of stainless steel NSSC 2120 and 2205 after 24 h interaction with a bacterial solution.

The SX-750 sample exhibited fewest colonies on a 24 h plate culture medium (Figure 10). The SX-650 and SX-950 samples exhibited relatively more colonies, but the difference between these samples was not significant. The SX-850 sample exhibited a large number of colonies on the plate culture medium. The colonies of each sample were counted and the R-value of each sample was calculated according to the formula of the antimicrobial rate in the Japanese industrial standard JIS Z:2801:2000. Table 3 summarizes the results.

After treating the SX-750 sample with the bacterial solution for 24 h, its antimicrobial rate was greater than 98% (Table 3). Considering the above standards and regulations, the samples aged at this temperature exhibited an antimicrobial effect. After the treatment of the SX-650, SX-850, and SX-950 samples and 2205 stainless steel with the bacterial solution for 24 h, their antimicrobial rates were 88.8%, 69.4%, 88.4%, and 0%, respectively. According to the evaluation, other aged specimens and 2205 stainless steel did not exhibit any antimicrobial effect. The difference in bacterial viability between the five samples above was significant, revealing that the antimicrobial effect is closely related to the size and distribution of the precipitated phase. A large number of rod-shaped ε-Cu phases precipitated at 750 °C, thereby increasing the surface area of the precipitated phase and rendering a better antimicrobial effect (Figure 4) [33]. In addition, the rod with a rich-Cu phase grew up to 300 nm. The copper-rich phase with rods growing up to 300 nm reportedly exceeded the thickness of the passivation film [34]; hence, the exposed ε-Cu phase on the surface did not form a passivation film. During sterilization, Cu^2+^ was formed by the action of the ε-Cu phase with water covering the passivation film, and the released Cu^2+^ was mainly sterilized by two routes: First, the cell wall was perforated when the Cu^2+^ penetrated to the bacteria and some intracellular substances leaked out, leading to bacterial death [35]. Second, when Cu^2+^ entered the microbial cells, it was bound to the enzymes in the bacteria and destroyed the enzymatic system in the bacterial cells. It can destroy the inherent components of microorganisms or produce functional disorders that affect the normal metabolism of bacteria, which leads to bacterial death. When the strain loses its activity, Cu^2+^ dissociates from the bacteria and sterilization is repeated. Hence, few colonies were observed on the plate culture medium of SX-750. Although SX-650, SX-850, and SX-950 did not exhibit an antimicrobial effect, they can kill certain bacteria for two reasons: On one hand, copper atoms bound to α-Fe were released to a certain extent after heat treatment, which afforded a small number of hydrated ions that exhibit a certain bactericidal effect. On the other hand, the solid-soluble copper also exhibited a certain bactericidal effect. SX-650 and SX-950 samples clearly exhibited higher antimicrobial rates than SX-850 because MnS precipitated from the SX-850 samples was soluble and easily formed CuS compounds. The cell walls and cell membranes contained negatively charged genes, and the formation of CuS led to a decrease in the restraint of metal cations on bacteria [36,37]; hence, the SX-850 samples exhibited the worst antimicrobial ability.

## 4. Conclusions

This study aims to obtain an underlying understanding of the effect of aging on the microstructure, mechanical properties, corrosion resistance, and antimicrobial properties of NSSC 2120 economical duplex stainless steel. The results obtained are useful for guiding the theoretical research and practical applications of the materials. The results are summarized as follows:

1. After aging NSSC 2120 stainless steel samples at various temperatures in the range 650–950 °C, γ and α phases were observed in its structure. In addition to these two phases, a ε-Cu phase was precipitated in SX-750, while CrMn_1.5_O_4_ and MnS phases were precipitated in SX-850.
2. The antibacterial rate of SX-750 was 98.8% due to the precipitation of the face-centered cubic structure of the ε-copper phase in the sample after aging at 750 °C, while simultaneously worsening the pitting resistance and decreasing the tensile strength of the material.
3. The oxides containing Mn and Cr (CrMn_1.5_O_4_) and sulfides (MnS) precipitated from the specimens after aging at 850 °C, the corrosion damage degree to the specimens was greater than that of the ε-Cu phase and the tensile strength of the specimens decreased to the lowest value; hence, the specimens did not exhibit an antimicrobial effect.

## Figures and Tables

**Table 1:** Chemical compositions of NSSC 2120 (mass fraction, %).

Element	C	Si	Mn	S	P	Cr	Ni	Mo	Cu	N	Fe
2120	0.007	0.100	3.269	0.007	0.014	20.942	2.265	0.435	1.322	0.182	Bal.

**Table 2:** Chemical composition of phase in the 2120 duplex stainless steel showed in Figure 4 (wt%).

Test Position	O	Ni	Si	Cr	Mn	Fe	Cu	S
①	0	0	0.27	18.8	3.33	58.57	17.99	0
②	0	0	0.26	21.46	3.13	67.06	7.36	0
③	28.53	0.01	0.12	47.56	20.78	1.82	0	0
④	0	1.03	0.24	22.28	5.36	67.53	1.32	0
⑤	1.07	0.01	0.48	1.05	65.46	2.72	0.14	29

**Table 3:** Antibacterial rates (R) of the samples after 24 h interaction with the bacterial solution.

Samples	Bacteria Count [cfu·mL^−1^]	R [wt%]
SX-650 °C	8.00 × 104	88.8
SX-750 °C	8.00 × 103	98.8
SX-850 °C	2.19 × 105	69.4
SX-950 °C	8.30 × 104	88.4
2205	7.15 × 105	0
